# Genomic Analysis of the Function of the Transcription Factor *gata3* during Development of the Mammalian Inner Ear

**DOI:** 10.1371/journal.pone.0007144

**Published:** 2009-09-23

**Authors:** Marta Milo, Daniela Cacciabue-Rivolta, Adam Kneebone, Hikke Van Doorninck, Claire Johnson, Grace Lawoko-Kerali, Mahesan Niranjan, Marcelo Rivolta, Matthew Holley

**Affiliations:** 1 Department of Biomedical Science, Addison Building, Western Bank, Sheffield, United Kingdom; 2 Department of Neurosciences, Erasmus Medical Centre, Rotterdam, The Netherlands; 3 Pfizer Global Research UK, Sandwich, Kent, United Kingdom; 4 Department of Electronics and Computer Science, University of Southampton, Southampton, United Kingdom; 5 NIHR Cardiovascular Biomedical Research Unit, Sheffield Teaching Hospitals NHS Trust, Sheffield, United Kingdom; University of Washington, United States of America

## Abstract

We have studied the function of the zinc finger transcription factor *gata3* in auditory system development by analysing temporal profiles of gene expression during differentiation of conditionally immortal cell lines derived to model specific auditory cell types and developmental stages. We tested and applied a novel probabilistic method called the gamma Model for Oligonucleotide Signals to analyse hybridization signals from Affymetrix oligonucleotide arrays. Expression levels estimated by this method correlated closely (p<0.0001) across a 10-fold range with those measured by quantitative RT-PCR for a sample of 61 different genes. In an unbiased list of 26 genes whose temporal profiles clustered most closely with that of *gata3* in all cell lines, 10 were linked to Insulin-like Growth Factor signalling, including the serine/threonine kinase *Akt/PKB*. Knock-down of *gata3* in vitro was associated with a decrease in expression of genes linked to IGF-signalling, including IGF1, IGF2 and several IGF-binding proteins. It also led to a small decrease in protein levels of the serine-threonine kinase Akt2/PKBβ, a dramatic increase in Akt1/PKBα protein and relocation of Akt1/PKBα from the nucleus to the cytoplasm. The cyclin-dependent kinase inhibitor p27^kip1^, a known target of PKB/Akt, simultaneously decreased. In heterozygous *gata3* null mice the expression of *gata3* correlated with high levels of activated Akt/PKB. This functional relationship could explain the diverse function of gata3 during development, the hearing loss associated with *gata3* heterozygous null mice and the broader symptoms of human patients with Hearing-Deafness-Renal anomaly syndrome.

## Introduction

Numerous regulatory genes have been implicated in the development of many tissues, including the inner ear [1], [2], [3], [4], but identification of the intracellular systems that they regulate remains a considerable challenge [5]. This challenge is compounded because a given gene may regulate several molecular events in different cell types during different developmental stages. Consequently, functional analysis should be done at cellular resolution during specified time windows. To achieve this in an organ as complex as the inner ear we have derived conditionally immortal epithelial and neuronal cell lines that have been characterised with a wide range of structural and functional markers with reference to their counterparts in vivo [6], [7], [8]. These cell lines can be differentiated in vitro and screened with whole genome oligonucleotide arrays to determine temporal profiles of gene expression. Assuming that functionally related genes are likely to share similar temporal expression profiles [9] we can explore such datasets to identify the influence that a given regulatory gene exerts on gene networks and intracellular systems. Whilst such information does not identify direct transcriptional targets it should provide valuable insight into the genetic regulation of cell behaviour.

The value of this approach depends very heavily on the rigor of the primary analysis of the array hybridisation signals. Given the biological variability and high level of noise in this kind of dataset we have developed probabilistic models, namely the gamma Models for Oligonuceotide Signals (gMOS), that provide a robust estimate of expression level along with a measure of uncertainty for each gene [10]. We have assessed the performance of gMOS on a biological dataset by re-analysing temporal expression profiles in an epithelial cell line derived from the cochlear duct of a mouse inner ear at embryonic day E13.5 [11]. Here we include new datasets from another epithelial cell line derived from the ventral otocyst at E10.5 that expresses key sensory cell markers during differentiation in vitro [6] and from one that expresses critical markers for cochlear neuroblasts [8].

We focus our analysis on the zinc finger transcription factor *gata3* [12], [13] for several reasons. First, it is an early regulator of auditory system development [14], [15] and is expressed in numerous cell types including sensory and non-sensory epithelial cells, efferent and afferent auditory neurons and periotic mesenchyme [14], [15], [16], [17]. Null mice die in mid embryonic development [12] and although a small otic vesicle forms it lacks a cochlear duct, semicircular canals and auditory ganglion [14]. There is direct evidence that gata3 regulates the expression of fibroblast growth factor 10 (*fgf10*) in the otic placode [18], [19]. Nevertheless, the expression patterns in vivo suggest that it has a more fundamental role in cell signaling [15]. All of the cell lines used in this work were selected for expression of *gata3* and provide an opportunity to explore its function at a cellular level. The second reason for studying *gata3* is that haploinsufficiency causes severe hearing loss in man [20], [21], [22] and heterozygous null mice suffer a hearing loss of about 30dB from the onset of hearing with a progressive, degenerative loss of sensory hair cells and spiral ganglion neurons [23]. This dose-dependency should increase the chances of identifying functionally associated genes by clustering temporal profiles of gene expression. Third, the expression of *gata3* is conserved amongst vertebrates and also occurs in T-lymphocytes, endothelial cells, placenta, kidney, adrenal gland, hair follicles and parts of the peripheral and central nervous systems [24], [25], [26], [27], [28]. Thus our results could be relevant to the development of many other tissues.

The results validate our application of gMOS models to real biological datasets from oligonucleotide arrays and have allowed us to establish entirely objective, ranked lists of genes clustered with *gata3* in each of 3 cell lines. A final list was prepared by selecting those genes that were most highly ranked in all cell lines under all culture conditions. The results reveal a previously unknown functional link to insulin-like growth factor signaling and to the serine/threonine kinase Akt/PKB, which is a hub signaling molecule for a number of tyrosine kinase receptors [29]. This may explain elements of the mechanism by which *gata3* might ‘orchestrate the regulation of different signaling pathways’ [26] and co-ordinate the development of the auditory system and other tissues, including the eye and central nervous system.

## Results

### Performance of gMOS and mgMOS for the analysis of gene expression data

Three main datasets were used in this study. They were taken from cells cultured for 14 days under differentiating conditions. The first included 12 time points for the epithelial cell line UB/OC-1 (OC-1), which was derived from organ of Corti at embryonic day E13.5 and cultured with 10% serum. This dataset is composed of 24 .CEL files with two chips (Affymetrix GeneChip® MU11k A and MU11k B) covering 11,000 genes of the mouse genome and originally analysed with Microarray Analysis Software MASv.5 [11]. The second dataset included 6 time points for the epithelial cell line US/VOT-E36 (VOT-E36), which was derived from the ventral part of the otic vesicle at E10.5 and cultured in serum-free media [6]. It was produced from 6 hybridisations to the Affymetrix GeneChip® Mg_U74Av2 for a total of six .CEL files. The third was a joint set taken only at 0 days and 14 days from VOT-E36 and US/VOT-N33 (VOT-N33) cultured with 10% serum [7] and hybridised on Affymetrix GeneChip® MU11k A and MU11k B (eight .CEL files). Whilst OC-1 and VOT-E36 represent epithelial cells at different developmental stages, VOT-N33 was derived from auditory neuroblasts at E10.5 [8]. The analysis was thus based upon 38 .CEL files representing 22 different cell preparations and three different array platforms. The datasets for the full temporal profiles have been deposited in the National Centre for Biotechnology Information (NCBI) Gene Expression Omnibus Database (http://www.nvbi.nlm.nih.giv/geo) under the accession numbers GSE36 and GSE15585.

We required a model that would estimate hybridization signals as accurately as possible with a robust estimate of the background, particularly those signals expressed at low levels, and that would provide a measure of uncertainty for each probe-set or transcript. The ability to do this also facilitates the need to merge data from different GeneChip platforms. Thus we chose to test the application of the gamma Model for Oligonucleotide Signals (gMOS) [10] and the subsequent modification, mgMOS [30], [31]. These probabilistic models provide estimates for the variance and credibility interval for each transcript and generate more accurate estimates of low levels of gene expression on benchmark datasets when compared with other stochastic models (Text S1). They were developed for the specific questions addressed in this study and have evolved into a more complete package for analysis of microarray data. They are now integrated into Bioconductor (http://www.bioconductor.org) in the package *puma* [32], an open source and open development software project for the analysis of genomic data.

**Figure 1:**
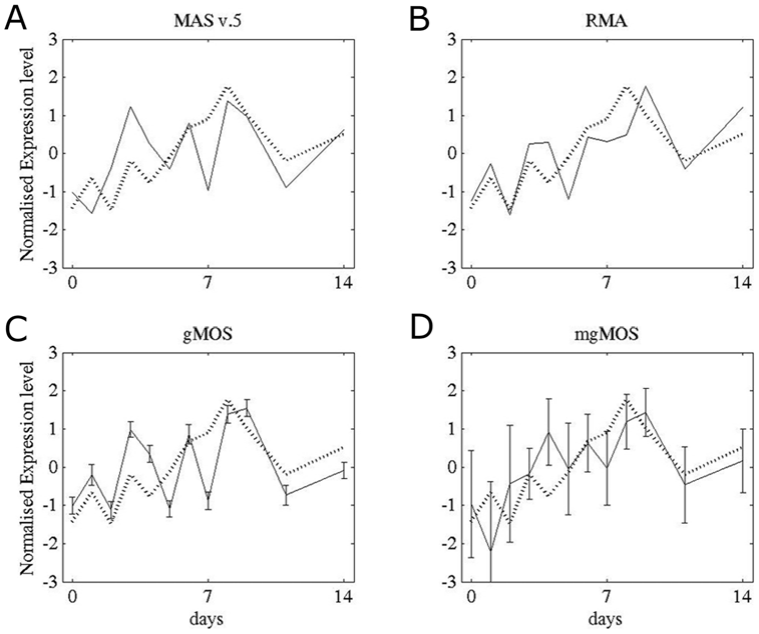
Temporal profiles for *gata3* in OC-1 cells calculated with four different models. We compared the expression profiles derived for gata3 from each model (solid lines) with the Taqman qRT-PCR profile (dotted line). To compare the data both the qRT-PCR values and the expression levels were standardised by subtracting the respective means and dividing by their standard errors. For gMOS and mgMOS we have plotted the variances evaluated for each time point by rejection sampling from the posterior distribution of the shape parameter α. A) MAS v.5, B) RMA, C) gMOS and D) mgMOS.

We compared the temporal expression profile for *gata3* derived by Taqman qRT-PCR from the cell line OC-1 with the same samples hybridized to the MU11k GeneChips and analysed with either gMOS, mgMOS, MASv.5 or Robust Microarray Analysis (RMA). The results from RMA were obtained with a single call of the algorithm in which all of the files were processed together. The qRT-PCR profile showed that *gata3* was expressed at very low levels before day 7, peaked at days 8–9 and then decreased to about half of the peak value by day 14 (Fig. 1). The presence of gata3 protein at all time points was confirmed both by immunocytochemistry and by immunoblotting. Signal detection from the array data through the first 7 days was variable. MAS v.5 did not preserve the RT-PCR profile for *gata3* (Fig. 1A). The RMA profile oscillated during the first week but the peak expression level was less than 2-fold higher than that at the start and not significantly better than the biological noise (Fig. 1B). gMOS did not detect a signal for the probe set at selected time points, which made the expression profile very irregular through the first 7 days, although it represented the peak as significant (Fig. 1C). mgMOS took account of the correlation between Perfect Match (PM) and MisMatched (MM) values on the arrays and provided a more accurate estimate than gMOS of low levels of expression as well as revealing the overall profile (Fig. 1D). The difference in the estimated level of gene expression between the first time points and the peak expression was about 5-fold which is substantially higher than the background noise. For each time point for both gMOS and mgMOS we plotted the variances evaluated by rejection sampling [33] from the posterior distribution of the parameters defining the gene expression signals. With mgMOS the variances were larger where the signal was low, a measure of uncertainty that was an important component of the subsequent clustering process.

**Figure 2:**
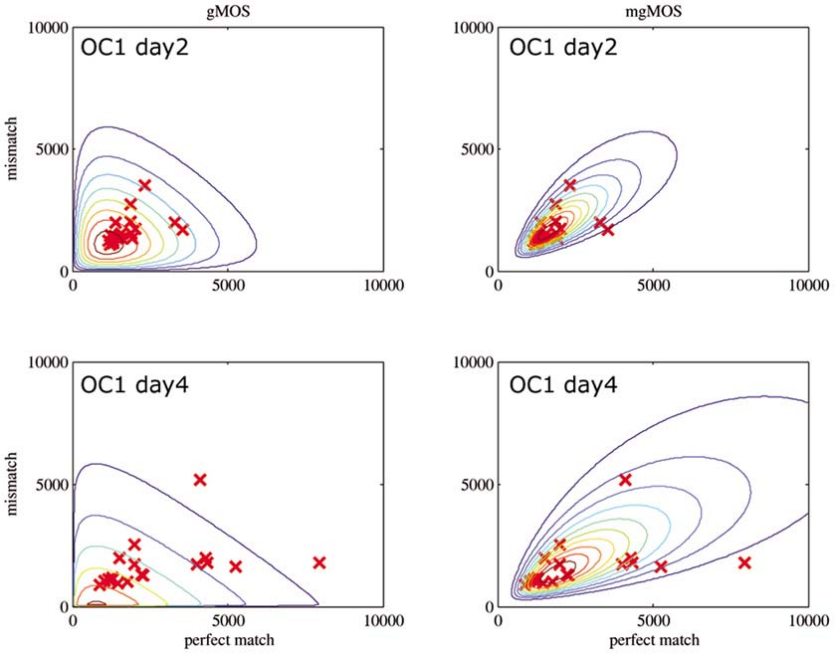
Contour plots to compare the performance of MASv5, RMA, gMOS and mgMOS. Contours of the likelihood functions of gMOS and mgMOS for the Perfect Match and MisMatch values of the *gata3* probe set for the cell line OC-1 at day 2 and day 4. To better assess the models we used Akaike's Information Criterion (AIC), the Bayesian Information Criterion (BIC) and the bias correction of AIC (AIC_C_ – See Table 1).

To further compare gMOS with mgMOS we plotted the contours of the likelihood functions for the PM and MM values of the whole dataset for days 2 and 4 and found that mgMOS fitted the data better than gMOS (Fig. 2). The more accurate performance of mgMOS was confirmed by calculation of Akaike's Information Criterion (AIC) [34] and the Bayesian Information Criterion (BIC) [35]. In this kind of data where the number of observations is smaller than 25, AIC has a tendency to overfit. To cover this issue, we also calculated the values of the bias correction of AIC (AICC) [36]. The results for all three information criteria (Table 1) showed that mgMOS was consistently better than gMOS across the full temporal profile. Thus mgMOS provided a better model for this type of data than MASv.5, RMA or gMOS and we used it in the next stage to analyse temporal expression profiles.

**Table 1:** Evaluation of AIC, AIC_C_ and BIC using OC-1 dataset.

Time Course	*gMOS AIC_C_*	*mgMOS AIC_C_*	*gMOS AIC*	*mgMOS AIC*	*gMOS BIC*	*mgMOS BIC*
day 0	708.88	**655.72**	706.88	**653.05**	709.87	**657.03**
day 1	706.24	**655.32**	704.24	**652.65**	707.23	**656.63**
day 2	716.75	**666.89**	714.75	**664.22**	717.73	**668.2**
day 3	737.72	**694.48**	735.72	**691.81**	738.71	**695.8**
day 4	713.97	**671.84**	711.97	**669.17**	714.96	**673.15**
day 5	706.34	**658.15**	704.34	**655.48**	707.33	**659.47**
day 6	705.72	**667.21**	703.72	**664.54**	706.71	**668.52**
day 7	693.88	**647.17**	691.88	**644.5**	694.87	**648.48**
day 8	717.85	**681.03**	715.85	**678.36**	718.83	**682.34**
day 9	717.83	**679.75**	715.83	**677.09**	718.81	**681.07**
day 11	675.76	**629.53**	673.76	**626.86**	676.75	**630.85**
day 14	741.91	**696.65**	739.91	**693.98**	742.9	**697.96**

Results for model selection by the Akaike's Information Criterion (AIC), the corrected Akaike's Information Criterion (AIC_C_) and Bayesian Information Criterion (BIC) using the *Gata-3* temporal profile data in OC-1. The bold data is associated with the better model.

### Analysis and hierarchical clustering of temporal expression profiles

Gene expression signals and associated uncertainties were extracted directly from the raw image (.CEL) files. Genes that shared similar expression profiles with *gata3* were grouped together by hierarchical clustering [37]. The precision measures obtained from mgMOS were used to smooth off noisy points and the similarity measures were calculated with respect to the differences between time points [38]. This was done to reduce fluctuation in the expression profile and thereby generate a more robust correlation measure. The approach is novel for gene expression data but is often used in time series analysis where a high rate of fluctuation in the data is expected. Both the difference approach in performing hierarchical clustering and the accuracy with which mgMOS estimated gene expression levels allowed us to generate robust, objective lists of genes clustered to *gata3*.

Temporal expression profiles were correlated with that of *gata3* in each cell line and ranked according to their Spearman correlation value. All genes with a correlation coefficient greater than 0.80 were then selected (Tables S1–S3). To gain a preliminary functional insight into our *gata3* clusters we analysed the representation of different gene families with the PANTHER (Protein ANalysis THrough Evolutionary Relationships: http://www.pantherdb.org) system [39]. We included all genes that were clustered to *gata3* with a correlation coefficient of at least 0.80 in all cell lines. In terms of Biological Processes significant representation (p<0.05) was found for cell proliferation and differentiation, apoptosis and intracellular signalling (Table 2). We then conducted a PANTHER Pathway analysis [40] to identify gene families associated with specific signalling pathways. Apoptosis, tyrosine kinase receptors (including FGF, PDGF and EGF) and integrin signalling were all represented in the top 10 pathways, consistent with previous studies on gata3 [18], [26].

**Table 2:** Biological process analysis of genes clustered to gata3 in silico.

Rank	Biological Process (from a total of 241 listed)
1	Cell proliferation and differentiation
2	Apoptosis
3	Protein metabolism and modification
4	Nucleoside, nucleotide and nucleic acid metabolism
5	Cell cycle
6	Cell cycle control
7	Intracellular signalling cascade
8	Sensory perception
9	Developmental processes
10	Transport

All genes clustered to *gata3* with a minimum correlation coefficient of 0.80 were analysed through the Panther system to compare their frequency of representation with that for the whole genome. The top ten cellular processes listed here, from a total of 241, were derived from the combined ranks from the three datasets.

### Core processes regulated by *gata3*

We assumed that the data from individual cell types would include genes that were directly and indirectly linked to *gata3* function in those cells. To identify genes that were more fundamentally linked to systems regulated by *gata3* we identified those that were common to all clusters and that shared correlation coefficients of >0.80. In this way we objectively established an unbiased list of 26 genes that correlated most closely with *gata3* in all of the datasets (Table 3). Ten of these genes were associated with insulin-like growth factor (IGF) signalling, including *Akt2*, *Igfbp7*, *Igfbpl1*, *MAP4K3* and *Ywhaz* (see discussion).

**Table 3:** Genes clustered to *gata3* in all cell lines and hybridizations.

Genes clustered to *gata3* in all cell lines and hybridisations	Gene Description
Akt2	thymoma viral proto-oncogene 2
Atp1a2	ATPase, Na+/K+ transporting, alpha 2 polypeptide
Clcn7	chloride channel 7
Cry1	cryptochrome 1 (photolyase-like)
Cryl1	crystallin, lamda 1
Cyp2b10	cytochrome P450, family 2, subfamily b, polypeptide 10
Dusp1	dual specificity phosphatase 1
Gas2	growth arrest specific 2
Gata3	GATA binding protein 3
Igfbp7	insulin-like growth factor binding protein 7
Igfbpl1	insulin-like growth factor binding protein-like 1
Itga6	integrin alpha 6
Map4k3	mitogen-activated protein kinase kinase kinase kinase 3
Mapk8ip3	mitogen-activated protein kinase 8 interacting protein 3
Mapt	microtubule-associated protein tau
Mlf2	myeloid leukemia factor 2
Mmp11	matrix metalloproteinase 11
Pitpn	phosphatidylinositol transfer protein
Pxmp3	peroxisomal membrane protein 3
Scn3a	sodium channel, voltage-gated, type III, alpha polypeptide
Serpind1	serine (or cysteine) proteinase inhibitor, clade D, member 1
Slc11a2	solute carrier family 11 (proton-coupled divalent metal ion transporters), member 2
Slc34a1	solute carrier family 34 (sodium phosphate), member 1
Sparcl1	SPARC-like 1 (mast9, hevin)
Tm4sf7	transmembrane 4 superfamily member 7 - tetraspanin 4
Ywhaz	tyrosine 3-monooxygenase/tryptophan 5-monooxygenase activation protein, zeta polypeptide

List of genes common to all cell lines that clustered to *gata3* with a minimum correlation coefficient of 0.80.

**Figure 3:**
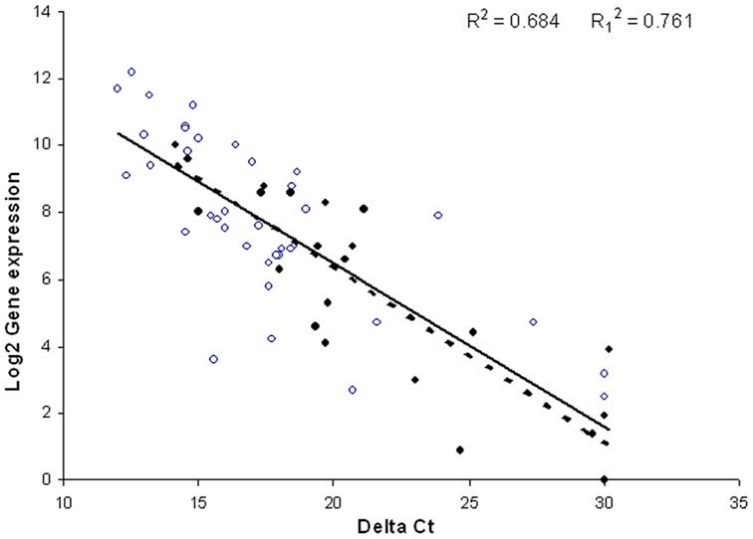
Correlation between gene expression levels calculated using mgMOS and those measured by Taqman RT-PCR. The correlation coefficient for the 61 genes represented here was significant (R^2^ = 0.684, p<0.0001), as was that for the 24 genes in the final cluster to *gata3* (R^2^ = 0.761, p<0.001). The relationship holds over a wide range of log_2_ values of gene expression from 0–12. The slope is the same for all genes (solid line for all points) and for those in the final cluster to *gata3* (dotted line for filled symbols only). The data show that mgMOS provides an accurate measure of gene expression for genes expressed at both high and low levels.

To validate this list and our estimates of gene expression level from the microarrays we assessed expression of 24 of the 26 genes in the final cluster to *gata3* by real time RT-PCR in the cell line VOT-E36 after 2 days of differentiation in vitro (Table 4). To strengthen this validation we included an additional 37 genes from outside the final cluster and for which effective probes were available. Given the association with PKB/Akt and IGF-signalling we selected the additional genes from these signalling pathways (Supplementary Table S4). There was a strong inverse correlation between the expression levels estimated by mgMOS and the ΔCT value from the RT-PCR (Fig. 3; n = 61, R^2^ = 0.7, p<0.0001). The range of values for absolute expression level was surprisingly close in terms of ‘fold change’ for the two methods. For example, *ywhaz* was expressed at a relatively high level with a log_2_ value of 10.0 and a ΔCT of 14.2, whilst *igfbp7* had a log_2_ value of 3.0 and a ΔCT of 23.0. These values represent a correlation across differences (fold changes) over the detection level of 2^7^–2^9^. This correlation provides strong experimental support for the application of mgMOS for estimating gene expression level from microarray data.

**Table 4:** qRT-PCR analysis of gene expression in VOT-E36 two days after differentiation in vitro.

Gene	Expression level in array for E36 at day 2 of differentiation in vitro	ΔCT - Taqman expression level for control	ΔΔCT - Change in expression after knock down of *gata3*	Summary comment
Akt2	9.1	16.6	−0.2±0.04	No change
Atp1a2	Not detected	Not detected	Not detected	Not detected
Cdkn1b*	7.5	16.1	−0.6±0.06	Down
Clcn7	8.6	17.3	0.1±0.09	No change
Cry1	7.0	20.7	−0.1±0.11	No change
Cryl1	5.3	19.8	−0.5±0.05	Down
Cyp2b10	1.9	Not detected	Not detected	Not detected
Dusp1	8.6	18.4	0.2±0.09	No change
Gas2	4.1	19.7	−0.4±0.09	No change
Gata3	9.6	14.7	−0.8±0.04	Down
Igfbp7	3.0	23.0	0.5±0.14	Up
Igfbpl1	1.4	Not detected	Not detected	Not detected
Itga6	8.0	15.1	0.5±0.03	Up
Map4k3	No probe	No probe	No probe	No probe
Mapk8ip3	6.6	20.4	0.1±0.03	No change
Mapt	4.6	20.7	−0.2±0.11	No change
Mlf2	8.8	17.4	0.3±0.05	No change
Mmp11	8.3	19.8	−0.4±0.06	No change
Pitpn	6.3	18.8	−0.7±0.07	Down
Pxmp3	8.1	21.2	−0.6±0.15	Down
Scn3a	Not detected	Not detected	Not detected	Not detected
Serpind1	4.4	24.9	−2.9±0.06	Down
Slc11a2	7.0	19.4	0.3±0.08	No change
Slc34a1	3.9	Not detected	Not detected	Not detected
Sparcl1	0.9	20.3	−0.5±0.01	Down
Tm4sf7	No probe	No probe	No probe	No probe
Ywhaz	10.0	14.2	−0.5±0.01	Down
Igf1	8.1	19.0	−0.7±0.02	Down
Igf1r	9.8	14.6	−0.1±0.08	No change
Igf2	12.2	12.5	−0.7±0.03	Down
Igfbp2	10.3	13.0	−1.5±0.07	Down
Igfbp3	7.8	15.7	−1.0±0.05	Down
Igf2bp3	Not detected	22.1	−1.6±0.06	Down
Irs1	2.7	20.7	−0.7±0.01	Down
Pik3cg	Not detected	31.0	2.4±0.50	Up
Foxo1	Not detected	13.0	−0.5±0.03	Down

The table includes the 26 genes from the final cluster to *gata3* and 9 from the IGF and Akt/PI3K signaling pathways for which we detected a significant change in expression following knock-down of *gata3*. *Cdkn1b (p27^kip1^) was not in the final cluster to *gata3* but is included here as a potential target of *PKB/Akt*. The correlation between expression levels from the arrays (column 2) and from Taqman analysis (column 3) for all 61 genes assessed by qPCR was highly significant and is shown in figure 3. Following knock-down of *gata3*, the expression levels of 9 of the 26 genes within the original *gata3* cluster changed significantly (7 down, 2 up, 10 unchanged, 7 undetected/no probe). Significant changes in expression were also observed for 8 genes outside the *gata3* cluster but in the IGF signaling pathway. These included *igf* and *igf2* but not *igf1r*. The threshold for change was set at a ΔΔCT value of 0.5 following subtraction of the variance.

The next task was to test the functional relationships between gata3, the genes listed in the final cluster and those associated with igf signaling. It was not possible to achieve a stable inactivation of gata3 in cell populations through equivalent temporal profiles. However, we were able to knock down *gata3* with siRNA at one time-point in a single cell line (Fig. S1) and to screen for changes in the same list of 61 genes analysed in the previous experiment. In terms of the original *gata3* cluster, 7 of the 26 genes were not detected by RT-PCR, 7 had lower expression levels in siRNA-treated cells, 2 had higher expression levels and 10 remained unchanged (Table 4). There was no measurable change in expression of the *igf1* receptor (*igf1r*) but there was a measurable down-regulation of genes in the IGF-signalling pathway, notably *igf1*, *igf2*, *igfbp2*, *igfbp3*, *igf2bp3* and *irs1*. *Pik3cg* was up-regulated. Considering the limitations of focussing on a single time-point and the fact that shared temporal expression profiles do not necessarily correlate with direct regulation, the observed changes provide clear evidence that gata3 is functionally linked to IGF-signaling. Because PKB/Akt was listed in the final cluster to *gata3* and it is the crucial hub signalling kinase in the IGF pathway [41] we chose to explore its functional links to gata3 in vitro and in vivo.

### PKB/Akt

*PKBβ/Akt2* is a serine-threonine kinase and the second of 3 mammalian isoforms, all of which were represented on the arrays. These kinases act as signalling hubs in a wide range of cellular functions from proliferation to growth, survival, apoptosis and differentiation [29]. They generally act downstream of receptor tyrosine kinases and it has been estimated that they potentially have up to 9000 downstream targets [42]. In the context of our original hypothesis that *gata3* regulates a core signalling pathway and in the light of our Panther system analysis, the potential link between *gata3* and *PKBβ/Akt2* was critical. Any influence of gata3 on the IGF pathway upstream of PKB/Akt should be reflected in the activation of these kinases.

**Figure 4:**
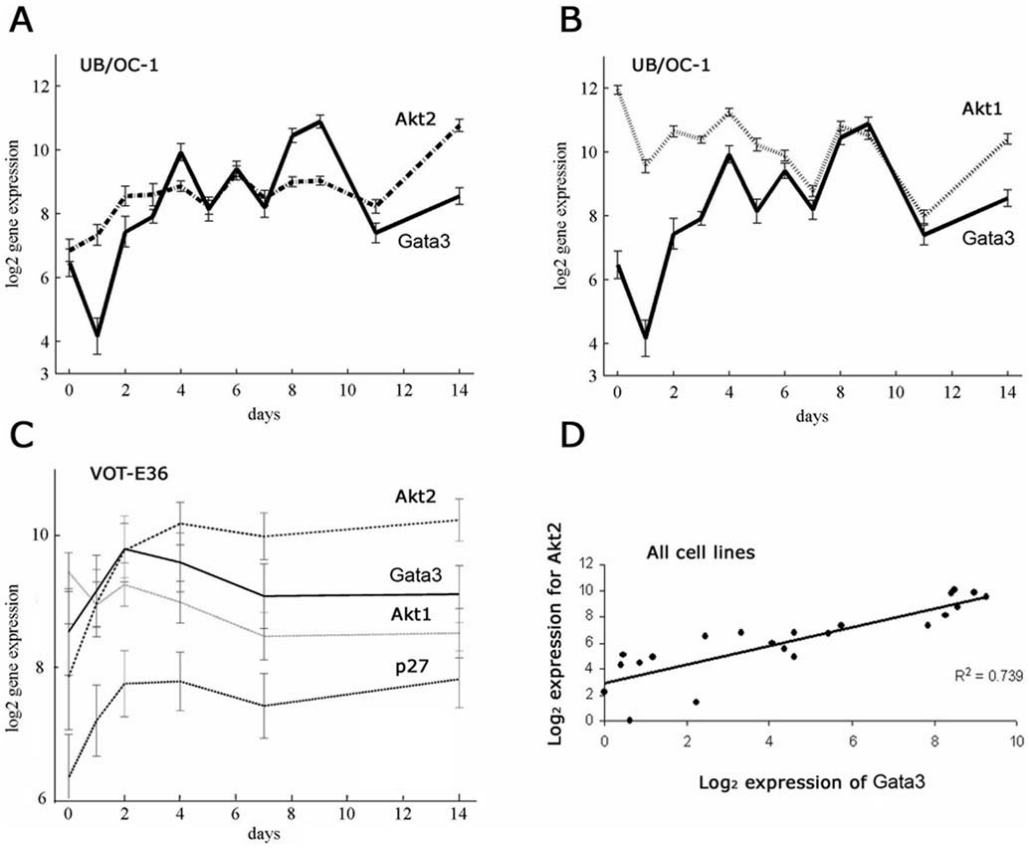
Expression profiles for *gata3* and *PKB/Akt* over a period of 14 days in vitro. OC-1 was cultured with serum and in the data represented here VOT-E36 was cultured in defined media. Cells were transferred to differentiating conditions at 39°C on day 0. All expression levels were plotted as log_2_ values in which each unit increase was equivalent to a 2-fold change. A – Expression of *gata3* and *PKBβ/Akt2* plotted against days of differentiation in OC-1 with serum. The shapes of the temporal profiles for the 2 genes were closely related after day 2. B - Expression of *gata3* and *PKBα/Akt1* plotted against days of differentiation in OC-1 with serum. C – Expression levels for *gata3, PKBβ/Akt2, PKBα/Akt1 and p27^kip1^* plotted against time for VOT-E36 in defined culture medium. D – Expression of *gata3* plotted against *PKBβ/Akt2* for all hybridizations, including data for VOT-N33 and VOT-E36 with serum. Each point represents a separate array, including 12 from OC-1, 6 from VOT-E36 in defined media, 2 from VOT-E36 in serum and 2 from VOT-N33 in serum. The correlation coefficient R^2^ was 0.739 (p<0.001).

In the array data the profiles for *PKBβ/Akt2* were highly correlated with *gata3* with respect both to shape and to absolute expression level. Those for *PKBα/Akt1* did not appear in the final list for all cell lines although the correlation in VOT-E36 without serum was ranked very close to that for *PKBβ/Akt2*. The signal profile for *gata3* in OC-1 showed an initial log_2_ expression value of about 6 at day 0, which increased by about 4-fold to a value of 10 at days 8–9 (Fig. 4A). There was then a 2-fold decrease by days 11 and 14. The equivalent profile for *PKBβ/Akt2* shared a similar absolute expression level and with the exception of the transition from day 0 to day 1, the fluctuations between successive time points of the temporal profile shared a similar pattern (Fig. 4A). The profile for *PKBα/Akt1* did not correlate as well with that for *gata3* until day 6 when they appeared to converge (Fig. 4B).

In VOT-E36 cultured in defined media the expression level for *gata3* was higher with a log_2_ value of 8–10 (Fig. 4C). The signal increased by 2–3-fold after 2 days of differentiation and then decreased slightly to 14 days. These expression levels were substantially higher than those obtained from the same cell line cultured with 10% fetal calf serum. However, the relative levels of expression between the 2 cell lines reflected those in the equivalent, native epithelial cells during normal development and were consistent with the high levels of gata3 protein in the ventral otic epithelium from E11–E18. The expression levels for *PKBβ/Akt2* were also higher in VOT-E36 than in OC-1 and the temporal profile was similar to that for *gata3* (Fig. 4C). The profile for *PKBα/Akt1* was also similar to that for *gata3*. In VOT-N33 cultured with serum *gata3* increased dramatically from 0–14 days, coinciding with a similar increase in *PKBβ/Akt2* (log_2_ = 2.1 to 8.0). This increase was consistent with results from immunohistochemistry on the cell line in vitro [43] and on sections of native cochlear neuroblasts in vivo [15].

The close correlation between *gata3* and *PKBβ/Akt2* was represented for all cell lines independently of the temporal profiles by plotting the expression levels of the 2 genes for all 22 array hybridisations (Fig. 4D). The correlation was highly significant (R^2^ = 0.739, p<0.001). The gene expression data thus indicated a positive correlation between *gata3* and *PKBβ/Akt2* across a 7-fold range of expression level and a lower but potentially significant correlation with *PKBα/Akt1*. Note that the correlation implies that in the absence of *gata3* there is significant expression of *PKBβ/Akt2*. *PKBβ/Akt2* is known to have a wider expression pattern than *gata3* and the observed correlation does not mean that it is solely or directly regulated by *gata3*.

### Effects of knocking down gata3 on PKB/Akt in vitro

**Figure 5:**
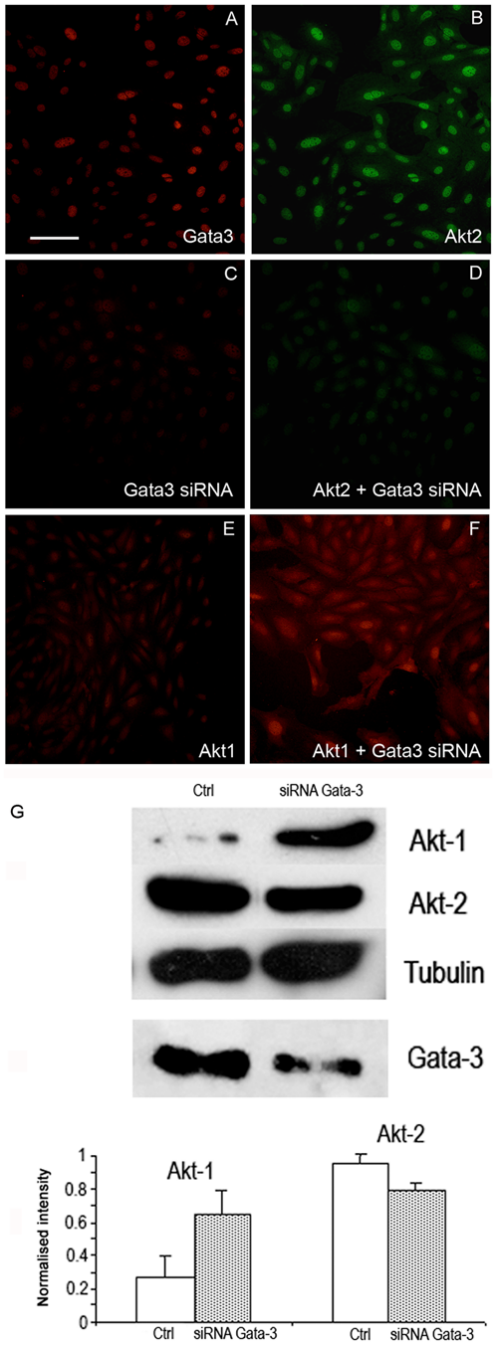
Immunofluorescence and immunoblotting for PKBβ/Akt2 and PKBα/Akt1 in VOT-N33 cultured with and without *gata3* siRNA. A–B – In untreated cells both gata3 and PKBβ/Akt2 were expressed in the cytoplasm and more obviously in the nuclei. C–D – Treatment with gata3 siRNA caused a loss of label for both gata3 and PKBβ/Akt2. E–F – In untreated cells PKBα/Akt1 was localized primarily to the nuclei but with siRNA to *gata3* the label was much higher and localized to the cytoplasm. Scale bar = 100 µm. G - Immunoblots for PKB/Akt in VOT-E36 after treatment with siRNA for *gata3*. The level of PKBα/Akt1 increased in the absence of gata3 whereas that for PKBβ/Akt2 decreased slightly relative to levels of tubulin. In the presence of siRNA gata3 was substantially but not completely knocked down (Fig. S1). Quantification of 6 different blots from 4 separate experiments revealed consistent evidence for the changes shown in A. (PKBα/Akt1 p<0.05; PKBβ/Akt2 p<0.02).

Immunofluorescence and immunoblotting revealed that gata3, PKBα/Akt1 and PKBβ/Akt2 were present in all of the cell lines. We tested the effects of knocking down *gata3* on PKBα/Akt1 and PKBβ/Akt2 by RNAi in the VOT-E36 cell line after 2 days of differentiation in vitro. Immunofluorescence labelling in both VOT-E36 and VOT-N33 cells showed similar results. Gata3 and PKBβ/Akt2 were co-localised in the nuclei of VOT-N33 cells (Fig. 5A–B) and the immunolabel for both was decreased when gata3 was knocked down (Fig. 5C–D). In contrast, there was a striking increase in the cytoplasmic label for PKBα/Akt1, which not only appeared to be up-regulated but also translocated from the nuclei (Fig. 5E–F). Immunoblotting revealed that lower levels of gata3 were associated with a small, reproducible decrease in PKBβ/Akt2 but in the same samples the levels for PKBα/Akt1 increased dramatically (Fig. 5G). This reciprocal change implied that both isoforms of PKB/Akt can be influenced by gata3 but that their overall levels, and by inference the levels of PKB/Akt activation, increase when gata3 is down-regulated.

### Expression of gata3, PKBβ/Akt2 and PKBα/Akt1 in the inner ear

**Figure 6:**
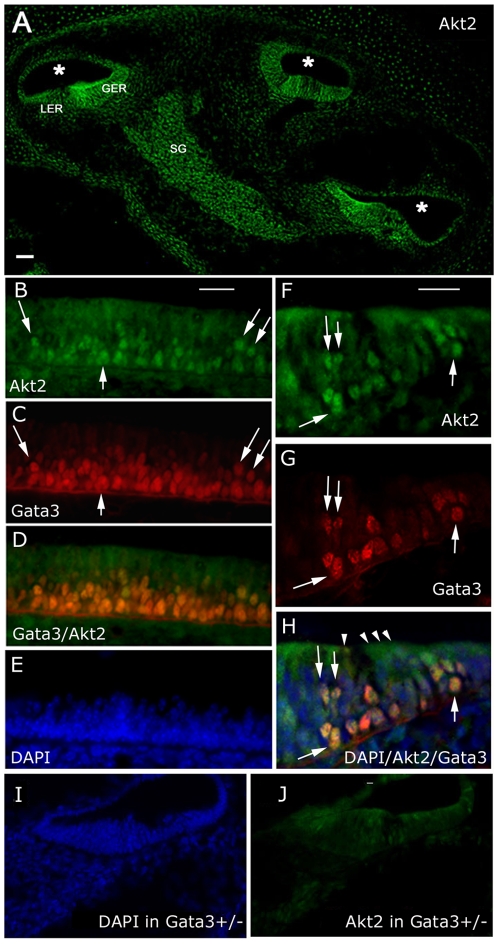
Expression of gata3 and PKBβ/Akt2 in E16.5 mouse cochlea. A – Immunofluorescence image of a cryosection through an unfixed, wild-type cochlea at E16.5 labeled with antibodies to PKBβ/Akt2. The spiral cochlear duct (*) was sectioned in 3 places. GER/LER – Greater/Lesser epithelial ridge, SG – Spiral ganglion. B,C,D,E - Immunofluorescence images of a longitudinal section along the organ of Corti. Where nuclear labeling for gata3 was high the label for PKBβ/Akt2 was also nuclear (arrows). Note that the top layer of cell nuclei labeled for DAPI (E) was very weakly labeled for gata3. F,G,H – Immunofluorescence images of a transverse section through the organ of Corti. Nuclei that labeled strongly for gata3 also labeled for PKBβ/Akt2 (arrows). Hair cell nuclei (cells indicated by arrowheads) were weakly labeled for gata3 and correspond to the top layer of cell nuclei in panel E. I,J - Immunofluorescence images of a transverse section through the organ of Corti from a heterozygous *gata3* null mouse. The images were prepared in parallel with those shown in panels F-H and indicate a lower level of label for PKBβ/Akt2. Scale bars = 100 µm.

If there is a relationship between gata3 and PKBβ/Akt2 in vivo then, despite the wider expression pattern of the latter, the two proteins should be co-expressed in sensory epithelia and spiral ganglion neurons during periods of high gata3 expression. In sections of the cochlea cut from normal mouse embryos at embryonic day E16.5, PKBβ/Akt2 was clearly expressed in the greater and lesser epithelial ridges as well as in the spiral ganglion (Fig. 6A). This labelling pattern overlapped that of gata3 at the same stage [17]. The antibody to gata3 was technically difficult to use because it was extremely sensitive to tissue fixation. However, double-labelling with antibodies to PKBβ/Akt2 did reveal co-expression in selected cells (Fig. 6B–H). In cells expressing high levels of gata3 the label for PKBβ/Akt2 appeared to be co-localised in the nuclei. The overall label for PKBβ/Akt2 was weaker in heterozygous *gata3* null mice (Fig. 6I–J) as was the label for gata3 (Fig. S2). More interestingly, the level of PKBβ/Akt2 in adjacent osteoblasts, which do not express gata3 but depend on PKBβ/Akt2 during differentiation [44], was unchanged in heterozygous gata3 null mice (Fig. S2). In heterozygous null mice colocalisation of gata3 and PKBβ/Akt2 was still evident in the cell nuclei but at lower levels (Fig. S3).

The cochlear expression pattern for PKBα/Akt1 did not correlate so well with gata3 (data not shown) and included many cells in the surrounding mesenchyme and connective tissue. Thus the immunofluorescence labelling in the native tissue correlated with the array analysis in the sense that there was a closer spatial relationship between gata3 and PKBβ/Akt2 than between gata3 and PKBα/Akt1.

### Activated PKB/Akt in heterozygous gata3 null mice

**Figure 7:**
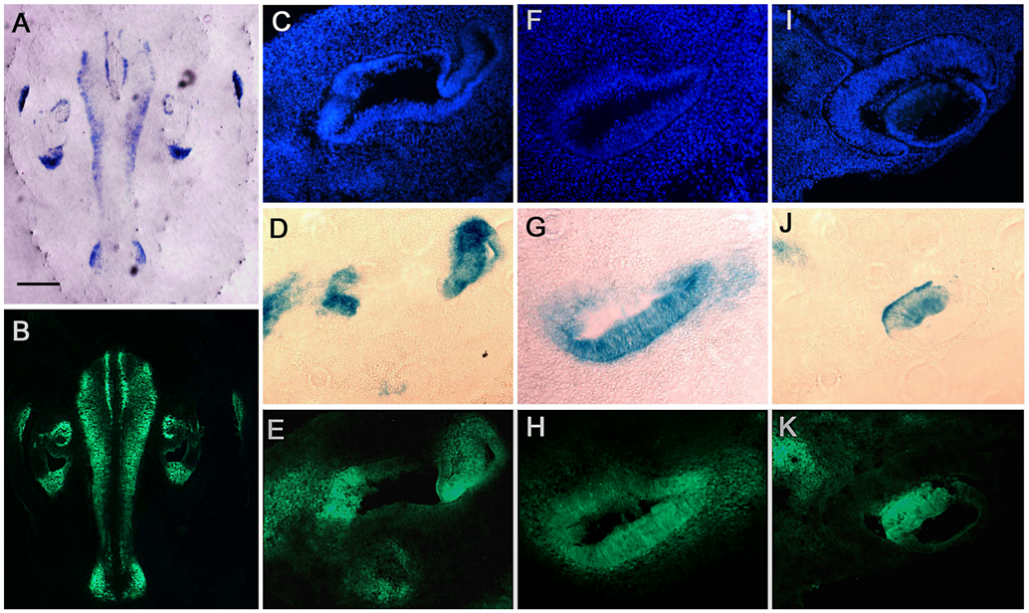
Correlation between expression of *gata3* in heterozygous null mice and immunolabelling for phosphorylated PKB/Akt. A - A section through the back of the head of a mouse at E11.5 showing regions expressing tau-LacZ in the hindbrain and in the ear. Scale bar = 500 µm. B – The same section as A labeled with antibodies to phosphorylated PKB/Akt. Note the similar labeling patterns in A and B. C,D,E – A higher magnification of a transverse section through the mid region of the inner ear showing tissue nuclei (Dapi), tau-LacZ and immunolabel for phosphorylated PKB/Akt, respectively. F,G,H – As C-E but for a section through the cochlear duct. I,J,K – As C–E but for a section through the eye and optic nerve (top left of each panel).

PKBβ/Akt2 was expressed in both heterozygous and homozygous *gata3* null mice. Expression in the latter is not surprising because PKBβ/Akt2 is not under the sole regulation of gata3. The otocyst in null mice remains cystic and it is not possible to identify the relevant cell types. Thus we were unable to derive meaningful information by analysing null animals. However, given that knock down of *gata3* led to only a small down-regulation of PKBβ/Akt2 but relatively large upregulation of PKBα/Akt1, as well as movement of PKBα/Akt1 to the cytoplasm of many cells, we predicted that overall levels of activated PKB/Akt should increase. This was reflected in *gata3* heterozygous null mice. To study this relationship we used antibodies to phosphorylated PKB/Akt although they do not distinguish the different PKB/Akt isoforms. For co-localisation with *gata3* we labelled heterozygous *gata3* null mice in which one *gata3* allele was replaced by a *tauLacZ* reporter gene. The lacZ expression pattern was correlated with high levels of activated PKB/Akt in all tissues and in all regions of the inner ear from E10.5 to E18.5 (Fig. 7). This relationship was also observed in the eye and the central nervous system. The data supports the previous evidence for a functional relationship between gata3 and PKB/Akt-signalling in epithelial, neuronal and mesenchymal tissues.

### Gata3, PKB/Akt and p27^kip1^

Gata3 has been linked to regulation of cell proliferation and survival [24] and p27^kip1^ (cdkn1b) is an important regulator of the cell cycle in cochlear sensory epithelial cells [45], [46] and sensory neurons [47]. p27^kip1^ is a known target of activated PKB/Akt, which can down-regulate its expression via FOXO transcription factors, inhibit its nuclear localisation and enhance its cytoplasmic degradation [29]. For these reasons we predicted that down-regulation of gata3 would lead to down-regulation of p27^kip1^. This prediction is relevant for the differentiation of hair cells which down-regulate both genes shortly after specification [17], [46].

**Figure 8:**
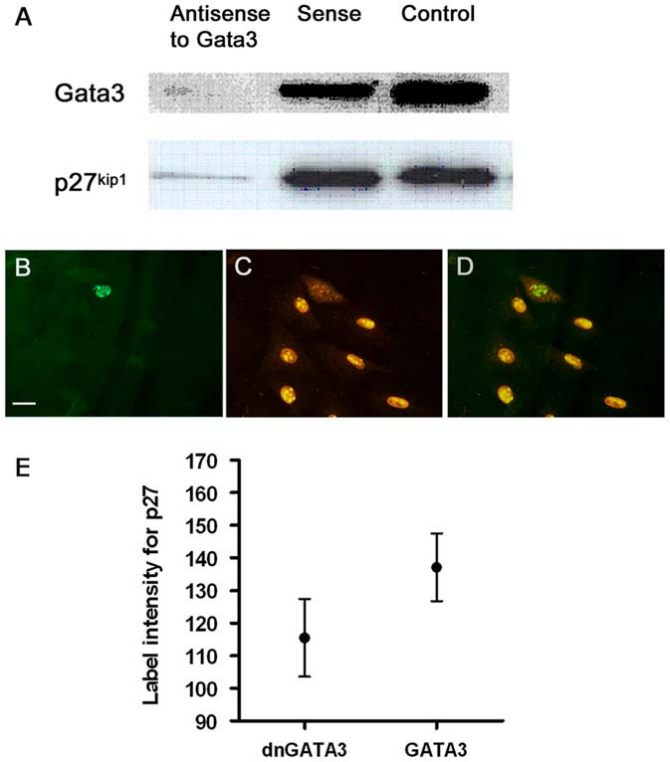
Regulation of p27^kip1^ by gata3 in the cell line VOT-N33. A – Antisense oligonucleotides used to knock down expression of *gata3* led to simultaneous loss of p27^kip1^ protein. B–D – These images are from the same group of cells. *dnGata3* (green label in B) led to cytoplasmic localisation of p27^kip1^ (labelled in red) and loss from the nucleus (C,D). E – In cells transfected with *dngata3* the levels of p27^kip1^ protein were significantly lower (p<0.0001). This result correlated with a decrease in expression of *p27^kip1^* (*cdkn1b*) following treatment with siRNA to gata3 (Table 4).

When *gata3* was knocked down with antisense oligonucleotides, p27^kip1^ was down-regulated (Fig. 8A) and its expression subsequently recovered as gata3 levels recovered. Furthermore, nuclear labelling for p27^kip1^ was low and often distributed to the cytoplasm in cells transfected with a dominant negative form of gata3 (Fig. 8B–E). Finally, the expression of p27^kip1^ decreased after cells were treated with RNAi for *gata3* (Table 4: ΔΔCT = −0.6±0.06 for cdkn1b).

## Discussion

This work shows that unbiased, statistical analysis of temporal gene expression profiles from appropriate cell lines can reveal novel insights into the function of regulatory genes during development. The high correlation between the signals derived from the microarrays and the Taqman RT-PCR shows that mgMOS is an effective probabilistic tool for analysis of the array hybridization signals and that it provides accurate estimates of gene expression level relative to equivalent data from quantitative RT-PCR. The analysis improved the detection of genes expressed at low levels and provided a measure of uncertainty for the expression level of each gene that was critical for ranking the correlations between temporal profiles. gMOS was developed to analyse the datasets presented in this paper and is now recognized as an extremely effective tool [32]. The results reinforce the value of the data analysis because they reveal a previously unknown functional association between gata3 and IGF-signalling, particularly via the hub serine/threonine kinase PKB/Akt.

### Association between gata3 and IGF-signalling

The top biological processes associated with genes clustered to *gata3* with a correlation coefficient of at least 0.80, including cell proliferation and differentiation, apoptosis, cell cycle and intracellular signalling, correlated well with those identified in a dataset based on differential gene expression profiles in hair follicles from wild type and conditional *gata3* null mice [26]. They are also consistent with the function of IGF-signalling, which is known to control cell survival, growth, proliferation and differentiation in both the auditory sensory epithelium and the spiral ganglion [48], [49], [50]. In terms of signalling pathways there was a high representation of genes associated with apoptosis and with several tyrosine kinase receptors. The link to fgf-signalling was relevant since gata3 regulates the expression of fgf10 in the otic placode [18] and mice lacking either fgf10 or gata3 have a much smaller otic vesicle [14], [19], which is associated with a decrease in cell proliferation [19]. *Fgf10* was represented on the arrays and was expressed in the cell lines as expected from the expression patterns from in situ hybridisation at the equivalent stage of development [18]. However, there was no obvious correlation with *gata3* in terms of the temporal expression profiles. Interestingly, the *gata3* clusters for the two epithelial cell lines included *fgfr2*, which encodes the receptor for fgf10.

The level of activity of PKB/Akt is critical during development to mediate IGF-signalling and the balance between cell proliferation, survival and growth [29], [41], [51]. However, many other genes listed in our final cluster to *gata3* are associated with IGF-signalling. The IGF-binding proteins Igfbp7 and Igfbpl1 interact directly with IGF, the former being widely expressed and binding IGFs specifically with relatively low affinity [52]. Ywhaz, also known as 14.3.3z, is a member of a family of proteins that mediate signal transduction by binding phosphoserine-containing proteins. It binds insulin receptor substrate 1 (irs1), thus influencing the sensitivity to insulin/IGF-signalling, but it also binds PKB/Akt directly [53] and can regulate the nuclear localisation of the cyclin-dependent kinase inhibitor p27^kip1^ [54]. *Irs1* appeared in the cluster to *gata3* in OC-1 with a correlation of 0.85. The dual specificity phosphatase Dusp1 is a protein tyrosine phosphatase with specificities for tyrosine and threonine and the ability to inactivate mitogen-activated protein kinase [55]. It is linked to cell proliferation and cell cycle control but also mediates apoptosis in rat pituitary tumour cells in response to thyroid hormone [56]. The phosphatidylinositol transfer protein, Pitpn, regulates intracellular transfer of phospholipids and the activity of phosphatidylinositol signalling pathways [57], [58].

The gene encoding the sodium/potassium ATPase subunit ATP1a2 also appeared in the final cluster to *gata3*. In vascular smooth muscle cells IGF-1 stimulates Na,K-ATPase via activation of PKB/Akt [59]. Ouabain inhibits Na,K-ATPases and can protect cultured kidney cells from apoptosis via activation of PKB/Akt [60]. Na,K-ATPases have a fundamental role in cellular homeostasis and may play a key role in neuronal cell death and neurodegeneration [61], [62], [63], thus their reduced function in *gata3* heterozygous null mice could account for the premature degeneration of the sensory epithelium. *ATP1a2* null mice suffer progressive hearing loss with minimal change in cochlear morphology [64]. Interestingly, these mice also have higher intracellular levels of chloride ions in their neurons mediated by malfunction of cation-chloride transporters [65] and hearing loss can be rescued by crossing the animals with those lacking the sodium/potassium/chloride transporter NKCC1 (SLC12a2) [64]. The presence of the intracellular chloride channel *Clcn7* in the *gata3* cluster is worth noting in this context.

Integrin α6 is dynamically regulated during neuroblast migration and epithelial differentiation in regions of the otocyst that express gata3 [66]. It's up-regulation in breast cancer cells leads to activation of PKB/Akt [67]. In prostate cancer cells IGF1 regulates expression of αvβ3 integrin via activation of PKB/Akt [68]. Thus the presence of Integrin α6 and Tm4sf7 (Tetraspanin 4), which forms complexes with Integrin α6 [69], may also be linked to IGF-signalling.

Finally, gata3 and PKB/Akt bind directly to smad3 [70], [71] and may thus modulate interactions between IGF and TGFβ-signalling pathways. The 3-phosphoinositide-dependent kinase PDK1, which phosphorylates PKB/Akt, also binds smad3 and is up-regulated with increased levels of IGF1 but down-regulated with higher levels of TGFβ [72]. Gata3 may thus regulate cell survival by priming the balance between these two pathways.

### Apoptotic pathways

Several proteins encoded by genes in the gata3 cluster apart from PKB/Akt are associated with apoptosis. The Cyp2 family of cytochrome p450 epoxygenases generate biologically active eicosanoids that act as second messengers in numerous signalling pathways, including those mediated by PKB/Akt, to regulate various processes including apoptosis [73]. *Cryl1* encodes a lambda-crystallin found in lens cells and that is highly expressed in liver and kidney [74]. Alpha-and beta-crystallins are known anti-apoptotic regulators in lens cells where their effects are mediated by the PKB/Akt and RAF/MEK/ERK pathways, respectively [75]. Mu-crystallin (Crym) is associated with gata3 in hair follicles [26], [76] and is linked to human deafness [77], [78]. Growth arrest specific protein Gas2 is a substrate of caspase-3 that sensitises cells to apoptosis in a p53-dependent manner [79]. MAP4K3 and MAPK8IP3 are involved in the JNK pathway, the activation of which facilitates apoptosis. In Drosophila, MAP4K3 is required for maximal activation of S6 kinase [80], which is part of a negative feedback to insulin/IGF receptor signalling [81], [82]. The matrix metalloproteinase Mmp11 mediates cell survival in epithelial cells via activation of PKB/Akt [83].

### Gata3 is functionally linked to activation of PKB/Akt

The diverse but highly conserved function of *gata3* [12] suggests that it regulates core elements of cell behaviour. Our results demonstrate a clear functional link to PKB/Akt although the association is complex. Although the gene array data indicated a high correlation between the expression levels of *PKBβ/Akt2* and *gata3* we detected little change in the expression of *PKBβ/Akt2* following knock-down of *gata3*. However, PKBβ/Akt2 was translocated from the nucleus to the cytoplasm with some evidence for down-regulation of the protein. An exclusive transcriptional link is unlikely because although the expression patterns for the two proteins were similar throughout the ear they were not identical and PKBβ/Akt2 was clearly expressed in groups of cells that lacked gata3. Nevertheless, nuclear localisation in vivo coincided with cells expressing high levels of gata3, consistent with the in vitro data. We conclude that the association was detected in the temporal profiles of gene expression because *gata3* and *PKBβ/Akt2* are simultaneously up-regulated and that they interact as proteins in a coherent functional pathway. The experimental approach was designed to identify intracellular processes associated with gata3 at physiological levels of expression rather than to identify direct transcriptional targets. Information derived from temporal profiles of gene expression cannot readily be compared with results from a knock-down of *gata3* in a single cell line at a specific time-point. The value of clustering temporal profiles depends on the assumption that the expression levels of functionally related genes change synchronously with time and that they reflect coherent cellular behaviours. In this context the data can potentially include transcriptional and protein-protein relationships.

The correlation between the expression of *PKBα/Akt1* and *gata3* was not as high as that for *PKBβ/Akt2* across all cell lines but was significant in VOT-E36 and from day 4 in OC-1 (Fig. 4). However, the effect of knocking down *gata3* at day 2 of differentiation in VOT-E36 was dramatic in terms of the up-regulation of PKBα/Akt1 protein and its translocation from the nucleus. The results show not only that gata3 regulates PKB/Akt but also that it has differential effects on PKBα/Akt1 and PKBβ/Akt2. Whilst we cannot conclude direct transcriptional control by gata3, when it was knocked down we observed changes in expression of several genes in the IGF-signalling pathway (Table 4), suggesting that there may be several ways in which changes in the expression of *gata3* could influence the activity of PKB/Akt.

### Gata3, PKB/Akt and IGF-signalling

Evidence for links between gata3, IGF-signalling and PKB/Akt in vivo come from studies on null mice. Gata3 is involved in the determination of skin cell lineages and when it is conditionally knocked down in the epidermis and hair follicles the animals suffer growth defects and dwarfism [26]. Pups are thinner with a substantial reduction in subcutaneous adipose tissue. This phenotype is similar to that described for mice lacking both PKBα/Akt1 and PKBβ/Akt2 with respect to dwarfism, impaired skin development and impeded adipogenesis [41]. It also closely resembles the phenotype of IGF-receptor null mice [84], prompting the conclusion that despite being a downstream effector of multiple growth factor receptors, PKBα/Akt1 and PKBβ/Akt2 are the most critical effectors of IGF signalling during development [41]. The striking similarities of these phenotypes reinforce the conclusions that we have drawn from an entirely objective gene expression analysis and suggest that the method provides an effective tool with which to explore the function of *gata3* and other regulatory genes.

## Materials and Methods

### Cell lines and cell culture

Three conditionally immortal cell lines were used for the gene expression analysis. UB/OC-1 (OC-1) was derived from auditory sensory epithelial cells at embryonic day E13.5, just prior to hair cell differentiation [85]. US/VOT-E36 (VOT-E36) was derived from epithelial cells in the ventral, auditory region of the otocyst at E10.5 and US/VOT-N33 (VOT-N33) was derived from auditory neuroblasts delaminating from the ventral otic epithelium at E10.5 [7]. All lines were selected for expression of gata3 protein.

The cells were cultured both in serum and serum free conditions. In serum VOT-E36, VOT-N33 and OC-1 were cultured in MEM (Invitrogen-GIBCO, Paisley, UK) and 10% fetal bovine serum (FBS) (Invitrogen-GIBCO, Paisley, UK) under immortalising conditions (33°C with γ-interferon (Peprotech Ec Ltd, London UK) and under differentiating conditions (39°C without γ-interferon). In serum free: VOT-N33 cells were cultured in Neurobasal Medium (NM) (Invitrogen-GIBCO, Paisley, UK) with 1% L-Glutamine (Invitrogen-GIBCO, Paisley, UK) and 2.5% FBS under immortalizing condition, and 2% B27 supplement (Invitrogen-GIBCO, Paisley, UK) under differentiating conditions. VOT-E36 cells were cultured in Ultraculture Medium (Cambrex-BioWhittaker, Europe) with 1% L-Glutamine. Flasks and dishes were coated with poly-D-lysine (Signa-Aldrich, UK) and fibronectin (Sigma-Aldrich, UK) was added to the medium just before plating.

### Gene array hybridization

The gene expression profiles were based on Affymetrix GeneChip® Mu11k (chip A and B) for the OC-1 cell line and GeneChip® Mg_U74Av2 arrays for VOT-E36 cell line.

Total RNA was prepared with Qiagen RNeasy mini-kits according to the manufacturer's instructions. cRNA was prepared as described in the Affymetrix GeneChip Expression Analysis Technical Manual. A comprehensive list of genes present in the chips and search tools are available at http://www.netaffx.com.

### Gata3 null mice

Homozygous and heterozygous *gata3* null mice in which the *gata3* alleles were replaced with a taulacZ reporter gene [24] were used to study co-expression with PKB/Akt and to study the effects of loss of function of gata3. They were bred from a cross between heterozygous parents to produce both types for comparison of pups from the same litter. Animal care and use was in accordance with the UK Home Office (Animal Procedures) Act 1986.

### Sections

C57/BL6 mice were killed by cervical dislocation in accordance with Home Office guidelines and the required tissue dissected out at 4°C in PBS. If PFA fixation was required then the tissue was immersed in 4% PFA in PBS for 2 hours at 4°C. The tissue was washed 3 times in PBS for 10 minutes per wash and cryoprotected in a 30% sucrose solution in PBS overnight at 4°C. Once the tissue had fully absorbed the cryoprotectant it was briefly dabbed dry and then frozen in Cryo-M-Bed embedding compound (Bright, UK) using liquid nitrogen cooled isopentane (BDH, Poole,UK). Embedded tissue blocks were stored long term at −80°C. Tissue samples were serially sectioned at 12 µm thickness onto gelatin/chrome-alum subbed slides with a cryostat (Bright,UK). Sectioned material was then stored at −80°C until required for processing.

### Antibodies, immunolabelling & microscopy

Cell culture media was aspirated from culture dishes and the cells fixed in either 4% Paraformaldehyde (PFA) (Sigma-Aldrich, UK) in Phosphate Buffered Saline (PBS) (Oxoid, UK) or a 1∶1 acetone/methanol mixture depending upon the primary antibody to be used. 4% PFA in PBS was immediately applied for 10 minutes. Acetone/methanol was applied for 2 minutes, before being aspirated and the cells allowed to dry for 5 to 10 minutes. After fixation, blocking solution consisting of 5% Goat Serum (Sigma-Aldrich, UK) in PBST was applied and allowed to block for 10 minutes. Blocking solution was removed and primary antibody in blocking solution applied at the required dilution. Primary antibodies used were mouse anti-GATA3 at 1∶50 (Santa Cruz Biotech, US), mouse anti-Akt1 at 1∶50 (Cell Signaling Technology, US), rabbit anti-Akt2 at 1∶150 (Cell Signaling Technology,US), rabbit anti Phospho-Akt (Thr308) (Cell Signaling Technology, Us) at 1∶100. Tissue culture dishes were placed into a humidified chamber and allowed to incubate with primary antibody for 2 hours. Primary antibody solution was then aspirated and cells washed 3 times in PBST for 5 minutes each wash. An FITC or TRITC tagged secondary antibody (Sigma-Aldrich, UK) raised against the appropriate animal was incubated with the cells for 1 hour in a blacked out and humidified chamber at a dilution of 1∶200 in blocking solution. Secondary antibody was aspirated and the cells washed 3 times in PBST for 5 minutes each wash. If required DAPI (4,6-Diamidino-2-phenylindole) (Sigma-Aldrich, UK) was added to the final PBST wash at a dilution of 1∶500 to allow for nuclear staining. The dishes were then mounted with Slowfade Gold (Molecular Probes Inc, US) mounting media and 32 mm glass coverslips (SLS, Scientific Laboratory Supplies, UK).

Sections were removed from storage at −80°C and allowed to thaw and air dry before being processed. A DAKO delimiting pen (DAKO Cytomation, Denmark) was used to form a barrier around each tissue section. Blocking solution consisting of 5% Goat Serum (Sigma-Aldrich, UK) in PBST was applied and allowed to block for 30 minutes. Sections were allowed to incubate in primary antibody (used at same concentrations as above) at 4°C over night. Primary antibody solution was then aspirated and cells washed 3 times in PBST for 5 minutes each wash. An FITC (Sigma-Aldrich, UK) or Alexa Fluor 568 anti-mouse IgG1(γ1) tagged secondary antibody (Molecular Probes, Invitrogen UK) was incubated for 1 hour in a blacked out and humidified chamber at a dilution of 1∶200 in blocking solution. Secondary antibody was aspirated and the sections washed 3 times in PBST for 5 minutes each wash. After the final PBST/DAPI wash the slides were mounted with Slowfade Gold mounting media and 22×64 mm glass coverslips (BDH, Poole, UK).

All visualization of immunohistochemistry was performed using a Zeiss Axiocam (Zeiss, US) attached to a Leica DMR microscope (Leica, Germany) connected to a computer running the Axiovision software (Zeiss, US).

### Immunoblotting

Protein extracts were prepared from cells growing at 33°C for 2 days and at 39°C for 1 to 7 days [86]. Cells from 80 cm^2^ flasks were lysed on ice adding 100 µl per flask of swelling buffer A (0.5M HEPES,1M KCl, 1M MgCl_2_, 1M DTT, 1M PMSF, Aproptin, Pepstatin A, Leupeptin and phosphatase inhibitors). Cells were allowed to swell on ice for 10 minutes and then centrifuged at 4°C at high speed for 3 minutes. Supernatant was saved for cytosolic proteins and stored at −80°C. The pellet was washed with 500 µl of buffer A and placed on 500 µl of buffer A with 47% sucrose and centrifuged at 15 RPM for 10 minutes. The pellet was than resuspended in 20–100 µl (according to the starting number of cells) of cold buffer C (20 mM HEPES, 25% glycerol, 420 mM naCl, 1.5 mM MgCl_2_, 0.2 mM EDTA, 1M PMSF, Aproptin, Pepstatin A, Leupeptin and phosphatase inhibitors) and incubated on ice for 10–20 minutes for high-salt extraction. Debris was removed by centrifugation and the supernatant stored at −80°C. Supernatants were assayed for protein concentration using the Bradford protein assay method. Samples containing 20–30 µg of protein were diluted with 4× sample buffer and boiled for 2–3 minutes before loading onto 7.5%–10% SDS PAGE gels. Proteins were transferred to nitrocellulose membranes using a semi-dry blotting system in 25 mM Tris, 192 mM glycine, 20% methanol and 0.1% SDS.

### Dominant negative gata3

Cells were seeded at a density of 6×10^4^ into 35 mm Nunclon tissue culture dishes (Nunc-Thermo Fisher Scientific, US) in low serum conditions consisting of Neurobasal media, 2.5% FCS, 1% gamma interferon and 1% L-glutamine and left to attach at 33°C for 24 hours. They were then washed with Neurobasal and transferred into serum free conditions consisting of Neurobasal media, 2% B27 supplement, 1% L-glutamine. Transfection with the gata3 KRR dominant negative DNA construct [87] was undertaken using Fugene6 (Roche, Burges Hill, UK) in accordance with the manufacturer's protocol. The cells were returned to 33°C for 24 hours to allow transfection to occur and then transferred to 39°C to allow differentiation to occur for up to 5 days.

### Gene silencing with RNAi

Gene silencing was performed using Silencer™ Pre-designed siRNA for gata3 from Ambion,Inc. (cat # 16704). Two different siRNA oligos were used (#61810 and #61725). The protocol for the transfection agent siPORT™ *Lipid* from Ambion, Inc. (cat # 4504) was optimised for each cell type used. Cells were transfected both in serum and in serum free conditions when shifted to 39°C. The transfection protocol suggested by Ambion for a 6-well plate was used in a 35 mm Petri dish, containing 1.5 ml of media. The cells were seeded at a density of 6.5–7.0×10^4^ in serum free media and at 6.0×10^4^ in serum. The transfection protocol was as follows: siPort lipid 6 µl (5 µl in serum free condition), 10 µl Opti-MEM (Invitrogen-GIBCO, Paisley, UK), siRNA 150 nM dissolved in Opti-MEM. The lipid port was incubated for 40 minutes and then mixed with siRNA dissolved in Opti-MEM. The complex was left to incubate for 40 minutes at room temperature before transfection. The efficiency of the transfection was checked with cy3 labelling of the siRNA using *Silencer™* siRNA Labelling Kit (Ambion Inc. cat # 1632). For transfection of 80 cm^2^ flasks the protocol was scaled up to 9 ml of media and cellular density to 6×10^5^ proportional to the increased surface of adherence and optimised to have more than 70% confluence after 24 h in culture.

The knock down of gata3 was tested with immunoperoxidase labelling, using primary antibody mouse anti-GATA3 at 1∶50 (Santa Cruz Biotech, US) and secondary biotinylated mouse antibody (Vector Laboratories, US) at 1∶200. Visualisation was achieved with a Vectastain ABC kit (Vector Laboratories, US) and peroxidase substrate kit (Vector VIP). The pixel intensities of 104 cells were measured in a control culture and in a siRNA culture. The cumulative distributions of the pixel intensities of both control and knock down cells were calculated. Normal probability plots and histograms were produced to check the distributions fit. Although both distributions were comparable the average pixel intensity in the control cells was 2.2 times higher than the knock down cells (Fig. S1).

### Gata3 phosphorothioate oligonucleotides

Antisense (AS) oligodeoxynucleotides were designed against the translational start region of GATA3 and consisted of 21-mer analogues [43]. A complimentary sense (S) sequence was designed and used as a control. Untreated cells (C) were also used as controls. The oligodeoxynucleotides were synthesised with a phosphorothioate backbone to improve resistance to endonucleases. The sequences, manufactured by MWGBiotech UK LTD, were as follows: GATA3 ‘AS’ DNA; 5′-CGC AGT CAC CTC CAT GTC CTC-3′; GATA3 ‘S1’ DNA; 5′-GAG GAC ATG GAG GTG ACT GCG-3′; and GATA3 ‘S2’ DNA; 5′-CTC CTG TAC CTC CAC TGA CGC-3′. Cells were cultured in 35 mm dishes or in 75 cm2 flasks in MEM/FCS and transiently transfected with various concentrations of either GATA3 AS or sense oligonucleotides mixed with Lipofectamine/Plus Reagent (Life Technologies, Gaithersburg, US) for up to 72 h at 33 and 39°C. Optimal concentrations were determined and the following concentrations selected: 1×10^5^ cells/35 mm dish, 0.4 µM GATA3 AS/S, 6 µl Lipofectamine, 6 µl Plus Reagent and 300 µl MEM or 5–8×10^5^ cells/75 cm^2^ flask, 1.5 µM GATA3 AS/S, 20 µl Lipofectamine, 20 µl Plus Reagent and 600 µl MEM. To assess the effect of the AS, GATA3 treated AS/S cells and non-treated cells were immunolabeled or immunoblotted with appropriate primary antibodies. Trypan blue staining was used to assess cell viability.

### TaqMan Low Density Arrays (TLDA)

Harvesting of cells and RNA extraction was carried out with an RNeasy Mini-Kit (Qiagen) according to the manufacturer's instructions. RNA was assessed and quantified spectrometrically and then stored at −80°C. cDNA was prepared with a High-Capacity cDNA Reverse Transcription Kit (Applied Biosystems) with 1 µg of total RNA for a final 20 µL reaction volume. After cooling on ice for 5 min, the cDNA was stored a −20°C. TaqMan probe and primer sets, including 18S rRNA for use as a reference, were selected from the Applied Biosystems web site following the UNIGENE accession number and/or Affymetrix probe set ID for each target. TaqMan Low Density Arrays (TLDAs) were custom designed on Applied Biosystems 7900HT 348-well 96a Micro Fluidic Card platforms. 5 µL of single-stranded cDNA (equivalent to 250 ng of total RNA) were mixed with 45 µL of nuclease-free water and 50 µL of TaqMan Gene Expression Master Mix (Applied Biosystems) and loaded into one sample port. The cards were centrifuged twice for 1 minute at 330 g and sealed to prevent well-to-well contamination. They were placed in the Micro Fluidic Card sample block on an ABI Prism 7900 HT Sequence Detection System (Applied Biosystems) following the “standard” thermal cycling option. Three controls with related RNAi cell cultures were used in duplicate on three different cards and the data analyzed together as a relative quantity (RQ) study. Expression values for target genes were normalized to the concentration of 18S rRNA. Estimates for gene expression were based on the comparative threshold cycle (Ct) method. The Ct data for all targets were used to evaluate the ΔCt values as (Ct_target_ - Ct_18S rRNA_) and then averaged across replicates. Standard deviation values were also calculated for each ΔCt. Subsequently, ΔΔCt values were calculated as ΔCt_target_ – ΔCt_calibrator_. The Control cultures were used as calibrators. The RQs were calculated as 2^− ΔΔCt^ and the SD for each ΔΔCt was calculated as (SD_1_^2^ + SD_2_^2^)^1/2^. Fold changes were calculated as –ΔΔCt±SD.

## Supporting Information

Text S1 Mathemathical details of the probabilistic models gMOS and mgMOS
(0.07 MB PDF)

Table S1 Genes clustered to Gata3 in OC-1 with a Spearman correlation of at least 0.800
(0.04 MB XLS)

Table S2 Genes clustered to Gata3 in VOT-E36 in serum-free media with Spearman correlation of at least 0.800
(0.17 MB XLS)

Table S3 Genes clustered to Gata3 in VOT-N33 and VOT-E36 cultured in serum, with a Spearman correlation of at least 0.800
(0.03 MB XLS)

Table S4 Correlation between gene expression level calculated by mgMOS and measured by qRT-PCR
(0.02 MB XLS)

Figure S1 Analysis of siRNA knock down of gata3 in vitro. Immunoperoxidase label for gata3 in a control culture and following treatment with siRNA to gata3 as described in the methods. The frequency histogram for intensity of nuclear labelling was derived from measurements made with ImageJ software. A measurable decrease in intensity was recorded in over 90% of the cell population.
(3.27 MB TIF)

Figure S2 Relative expression of gata3 and akt2 in gata3+/+ and gata3+/− mice. Sections through cochlear ducts of gata3+/+ and gata3+/− mice at E16.5 double-labeled with antibodies to gata3 and akt2. The images were exposed and reproduced in parallel under identical conditions. In gata3+/− mice the label for both gata3 and akt2 was less intense in most cells in the cochlea. The lower left panel confirms the overlap between the two labels in gata3+/+ mice. The lower right panel shows the distribution of cell nuclei in gata3+/− mice, highlighting the fact that the few cells expressing detectable levels of gata3 also labelled for akt2 (arrowheads). Note that the label for akt2 in the cochlear cartilage (asterisk) was the same for both animals. Akt2 is expressed in many different cell types, including chondrocytes, but levels were unaffected in gata3+/− mice in cells that did not normally express gata3. Scale bar = 100 µm
(5.34 MB TIF)

Figure S3 Expression of gata3 and akt2 in gata3+/− mice. Sections through cochlear ducts of gata3+/− mice at E16.5 double-labeled with antibodies to gata3 and akt2. The antibodies to gata3 were sensitive to tissue fixation and were used at high concentration in gata3+/− mice to detect the low levels of gata3. This caused non-specific label in the basement membrane. Nevertheless, in separate sections of the cochlea in gata3+/− mice there was a clear overlap between gata3 and akt2, despite the much lower expression of both proteins compared to that in normal mice. Scale bar = 100 µm
(2.98 MB TIF)
