# Supplementary material for: Genomic Analysis of the Function of the Transcription Factor gata3 during Development of the Mammalian Inner Ear
Source: PLoS One. 2009 Sep 23;4(9):e7144. doi: 10.1371/journal.pone.0007144 (PMC2742898; doi:10.1371/journal.pone.0007144)
Supplement: Text S1 — Mathemathical details of the probabilistic models gMOS and mgMOS (0.07 MB PDF) [file pone.0007144.s001.pdf]

Mathemathical details of the probabilistic models gMOS and mgMOS

gamma Model for Oligonucleotide Signal (gMOS)

gMOS reflects the fact that both the perfect match (PM) and mismatch (MM) intensities are positive and makes an assumption that the estimated gene expression signal is constrained to be positive. The model also assumes that PM and MM are independently sampled and therefore they are drawn from two independent probability distributions. PM is represented by a random variable ***y***, ***m*** is a random variable representing MM and ***s*** is the random variable representing the gene expression signal. The variables in the model are y_{ij}, m_{ij} and s_{ij} with *i=1,…,n_j* and *j=1,…,N,* where n_j is the number of probe pairs in the *j*th probe set and *N* is the total number of probe sets on the chip. Under the above hypothesis of positive definition of the variables, if *m* and *s* are gamma distributed with shape parameters *a* and α respectively and same scale parameter *b*, their sum is still a gamma distributed variable with shape parameter $\alpha + a$ and scale parameter *b*. Therefore, if $m_{ij} \sim Gamma(a_j, b_j)$, $y_{ij} \sim Gamma(\alpha_j + a_j, b_j)$, $s_{ij} \sim Gamma(\alpha_j, b_j)$ and we can derive the following probabilities:

$$p(m_{ij} \mid a_j, b_j) = \frac{b_j^{a_j}}{\Gamma(a_j)} m_{ij}^{a_j - 1} \exp(-b_j m_{ij}),$$

$$p(s_{ij} \mid \alpha_j, b_j) = \frac{b_j^{\alpha_j}}{\Gamma(\alpha_j)} s_{ij}^{\alpha_j - 1} \exp(-b_j s_{ij}),$$

$$p(y_{ij} \mid a_j + \alpha_j, b_j) = \frac{b_j^{\,a_j+\alpha_j}}{\Gamma(a_j + \alpha_j)} y_{ij}^{a_j+\alpha_j-1} \exp(-b_j y_{ij})$$

where $\Gamma(\cdot)$ is the gamma function. To define the distributions we need to estimate the parameters α_j, a_j and b_j. For this purpose, we derived the joint log-likelihood function, $L(a_j, \alpha_j, b_j) = L(a_j, b_j) + L(\alpha_j + a_j, b_j)$, where

$L(a_j, b_j) = \log(\prod_{i=1}^{n_j} p(m_{ij} \mid a_j, b_j))$ and $L(a_j + \alpha_j, b_j) = \log(\prod_{i=1}^{n_j} p(y_{ij} \mid a_j + \alpha_j, b_j))$, and maximize it with respect to the parameters α_j, a_j and b_j. To solve this optimization problem we used the scaled conjugate gradient algorithm (1). Once the parameters are estimated we can calculate the expected probe signal $< s_j >$ and the associated precision $1/\sigma_j^2$ as mean and variance, respectively, of the gamma distributed variable ***s***. They are respectively:

$$< s_j > = \frac{\alpha_j}{b_j} \quad \text{and} \quad \sigma_j^2 = \frac{\alpha_j}{b_j^2}.$$

In our experiments we used the log of the gene expression signals and therefore we calculated the expected value and the variance of the transformed variable log(***s***). They can be defined as $< \log(s_j) > = \psi(\alpha_j) - \ln(b_j)$ and $\sigma^2_{\log(s_j)} = \psi'(\alpha_j)$,

where $\psi(\alpha_j) = \frac{\partial}{\partial \alpha_j} \log(\Gamma(a_j))$ and $\psi'(\alpha_j) = \frac{\partial}{\partial \alpha_j} \psi(\alpha_j)$.

Modified gamma Model for Oligonucleotide Signal (mgMOS)

In modified gMOS (mgMOS) the assumption that the PM and MM intensities are positive and the signal is constrained to be positive is still in place but the PM and MM are no longer assumed to be independently sampled. In mgMOS we aim

to model the correlation that is empirically observed between PM and MM. This correlation is particularly strong for probes with relative low signal. Under the above assumptions the variables ***y*** and ***m*** are drawn from a joint probability function and no longer from two independent distributions. Thus we have

$$p(y_{ij}, m_{ij}) = \int p(y_{ji} \mid a_j, \alpha_j, b_{ij}) p(m_{ij} \mid a_j, b_{ij}) p(b_{ij}) db_{ij}$$

where $b_{ij} \sim Gamma(c_j, d_j)$. The parameters b_{ij} reflect the different binding affinity of probes within the probe set. In the original model gMOS the binding affinity is assumed not to vary within the probe set and therefore the model does not take into account the effect on the Gene Specific Binding (GSB) signal of the homomeric base change in the MM probes (2). The new model mgMOS, instead, is designed to capture this effect by introducing an additional level of complexity on the parameter ***b***. To model the BA as varying within the probe set mgMOS allows the parameter ***b*** to assume different values for each probe pair and ***b*** is therefore drawn from a probability distribution that influences the estimation of the signal ***s***. If this probability distribution is a *gamma* distribution, as are the probability distributions of ***y*** and ***m*,** then the integral in the above equation is tractable and the estimate of ***s*** can be computed analytically. Thus, the resulting distribution of the gene expression signal ***s***, given the above constraints, has the following form:

$$p(s_{ij} \mid \alpha_j, c_j, d_j) = \int p(s_{ij} \mid \alpha_j, b_{ij}) p(b_{ij}) db_{ij} = \frac{d_j^{c_j} s^{\alpha_j + 1} \Gamma(\alpha_j + c_j)}{\Gamma(\alpha_j)\Gamma(c_j)(s_j + d_j)^{\alpha_j + c_j}}.$$

The parameters α_j, a_j, c_j and d_j are estimated, as for the gMOS model, by maximizing the log likelihood function $L(a_j, \alpha_j, c_j, d_j) = \log(\prod_i p(y_{ij}, m_{ij}))$ using a

scaled conjugate gradient algorithm. A disadvantage of the mgMOS approach is that the log likelihood is no longer unimodal with respect to the parameters (as it was for the gMOS algorithm). In our experiments we always initialised the model by setting $\alpha_j = a_j = c_j = d_j = 1$. The expected probe signal and its variance are respectively given by:

$$< s_j > = \frac{\alpha_j d_j}{c_j - 1} \text{ and } \sigma_j^2 = \frac{c_j^{\ 2}(c_j + \alpha_j - 1)}{\alpha_j (c_j - 1)^2 (c_j - 2)}.$$

Similarly we can calculate the expected value and the variance of the transformed variable *log(**s**)*. They are respectively defined as

$< \log(s_j) > = \psi(\alpha_j) - \psi(c_j) + \log(d_j)$ and $\sigma^2_{\log(s_j)} = \psi'(\alpha_j) + \psi'(c_j)$. In both gMOS and mgMOS it is possible to derive the posterior distribution of the parameters α_i as an approximation by a Gaussian distribution whose mean corresponds to a Maximum A posteriori (MAP) estimate under an uniform prior on α_i and the variance corresponds to the curvature of the log-likelihood L as function of α_i. The MAP estimate and the curvature are evaluated for the Maximum Likelihood estimates of the parameters.

References:

1. Zhang L, Miles MF, Aldape KD (2003) **A Model of molecular interactions on short oligonucleotide microarrays.** Nat Biotech 21: 818-821.

2. Nabney,I.T.(2001). **NETLAB:Algorithms for Pattern Recognition**. Springer Series: Advances in Pattern Recognition. Springer-Verlag, London.
