# Supplementary figures and images for: Genomic Analysis of the Function of the Transcription Factor gata3 during Development of the Mammalian Inner Ear
Source: PLoS One. 2009 Sep 23;4(9):e7144. doi: 10.1371/journal.pone.0007144 (PMC2742898; doi:10.1371/journal.pone.0007144)

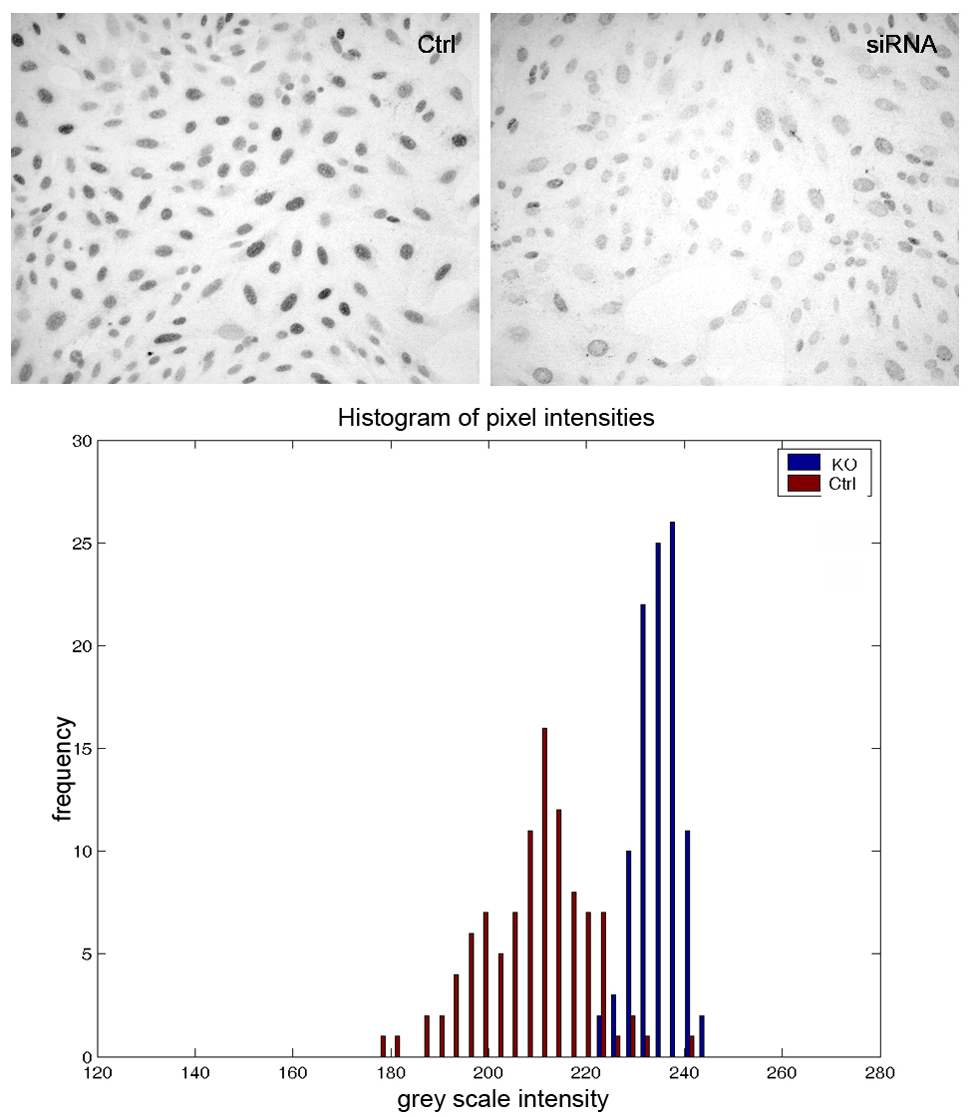

Supplement: Figure S1 — Analysis of siRNA knock down of gata3 in vitro. Immunoperoxidase label for gata3 in a control culture and following treatment with siRNA to gata3 as described in the methods. The frequency histogram for intensity of nuclear labelling was derived from measurements made with ImageJ software. A measurable decrease in intensity was recorded in over 90% of the cell population. (3.27 MB TIF) [file pone.0007144.s006.tif]

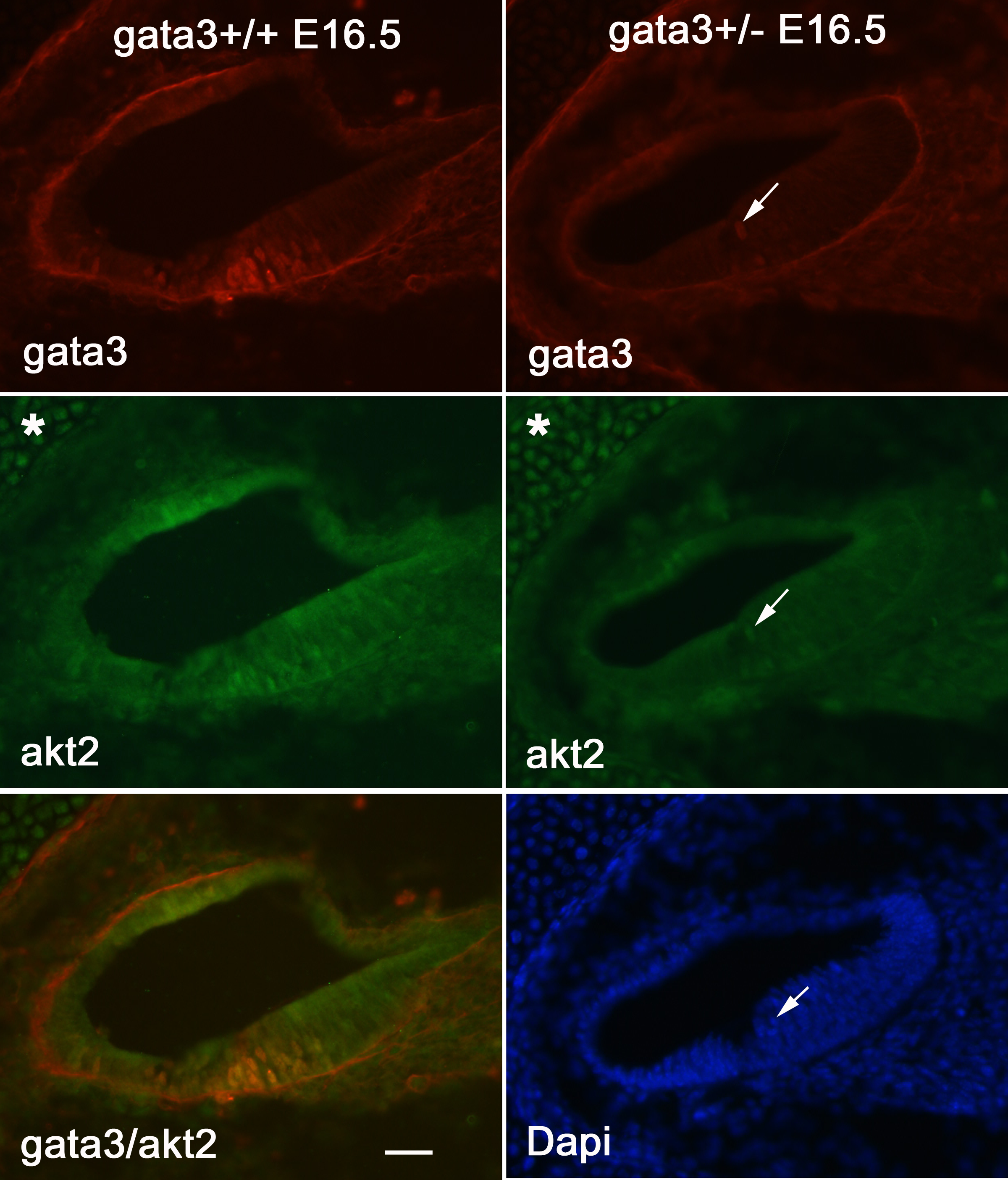

Supplement: Figure S2 — Relative expression of gata3 and akt2 in gata3+/+ and gata3+/− mice. Sections through cochlear ducts of gata3+/+ and gata3+/− mice at E16.5 double-labeled with antibodies to gata3 and akt2. The images were exposed and reproduced in parallel under identical conditions. In gata3+/− mice the label for both gata3 and akt2 was less intense in most cells in the cochlea. The lower left panel confirms the overlap between the two labels in gata3+/+ mice. The lower right panel shows the distribution of cell nuclei in gata3+/− mice, highlighting the fact that the few cells expressing detectable levels of gata3 also labelled for akt2 (arrowheads). Note that the label for akt2 in the cochlear cartilage (asterisk) was the same for both animals. Akt2 is expressed in many different cell types, including chondrocytes, but levels were unaffected in gata3+/− mice in cells that did not normally express gata3. Scale bar = 100 µm (5.34 MB TIF) [file pone.0007144.s007.tif]

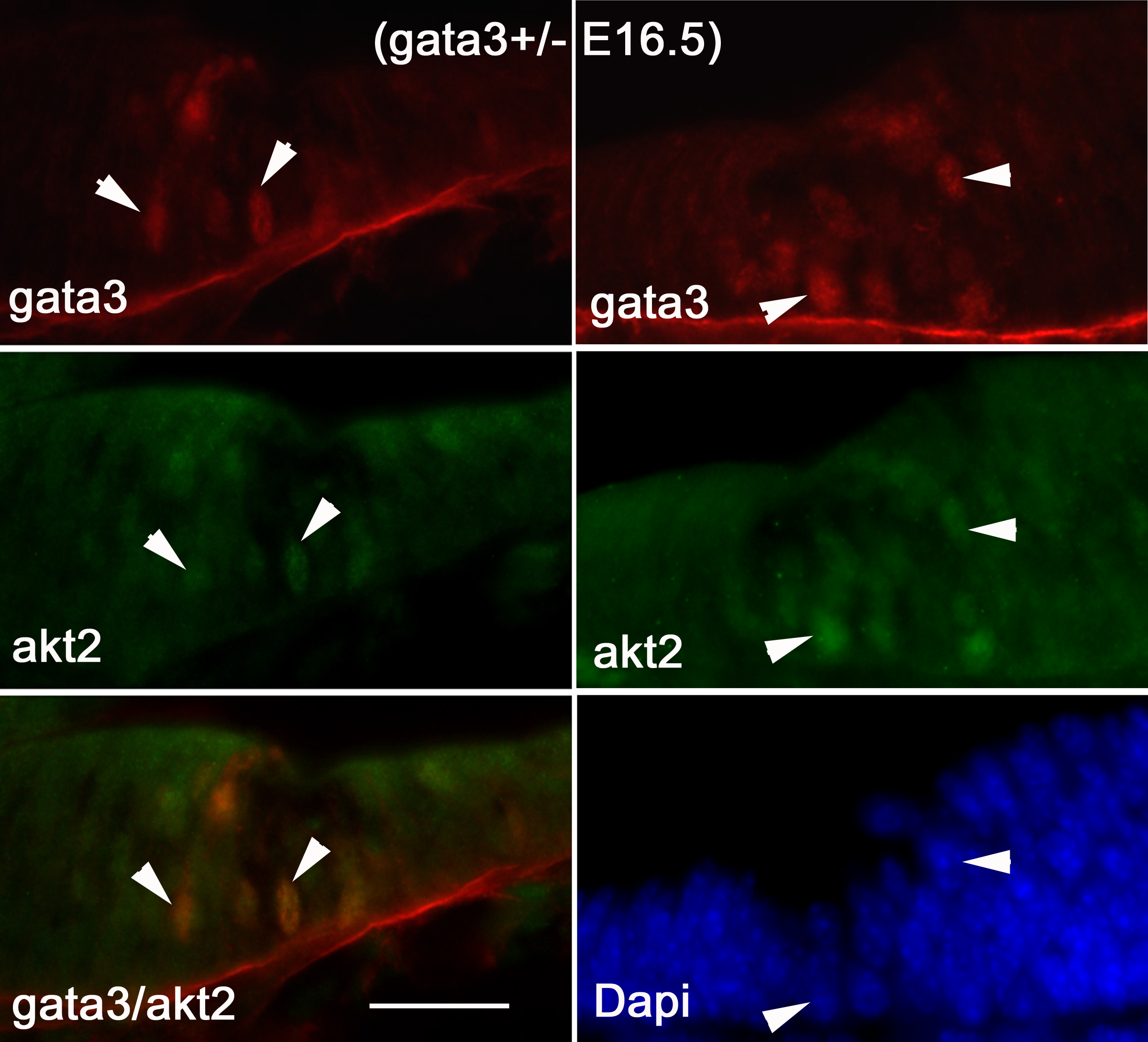

Supplement: Figure S3 — Expression of gata3 and akt2 in gata3+/− mice. Sections through cochlear ducts of gata3+/− mice at E16.5 double-labeled with antibodies to gata3 and akt2. The antibodies to gata3 were sensitive to tissue fixation and were used at high concentration in gata3+/− mice to detect the low levels of gata3. This caused non-specific label in the basement membrane. Nevertheless, in separate sections of the cochlea in gata3+/− mice there was a clear overlap between gata3 and akt2, despite the much lower expression of both proteins compared to that in normal mice. Scale bar = 100 µm (2.98 MB TIF) [file pone.0007144.s008.tif]
